# Predictors of cartilage degeneration in patients with subchondral insufficiency fracture of the femoral head: a retrospective study

**DOI:** 10.1186/s13075-020-02243-7

**Published:** 2020-06-22

**Authors:** Tomohiro Shimizu, Shunichi Yokota, Yosuke Kimura, Tsuyoshi Asano, Hirokazu Shimizu, Hotaka Ishizu, Norimasa Iwasaki, Daisuke Takahashi

**Affiliations:** Department of Orthopaedic Surgery, Faculty of Medicine and Graduate School of Medicine, Hokkaido University, Kita-15 Nishi-7, Kita-ku, Sapporo, 060-8638 Japan

**Keywords:** Subchondral insufficient fracture, Joint space narrowing, Hip joint, Bone metabolic marker

## Abstract

**Background:**

There is evidence that the cause of primary osteoarthritis (OA) is related to the changes in subchondral bone; however, the influence of subchondral insufficiency fracture (SIF) of the femoral head on the degeneration of the hip joint and the prognostic factors related to joint degeneration remain unclear. The objectives of this study were (1) to investigate the natural history of joint space width after the occurrence of SIF and (2) to investigate the associations between joint space narrowing and bone metabolic markers as well as magnetic resonance imaging (MRI) among the patients with SIF.

**Methods:**

Between January 2010 and December 2019, 238 patients in whom band pattern of the femoral head were observed on MRI visited Hokkaido University Hospital. Among these patients, 44 hips in 41 patients were diagnosed with SIF and eligible for this retrospective study. We evaluated the joint space width (JSW) of the hip on the radiograph obtained at the first and last visits, length of the band lesion on MRI, bone mineral density by dual-energy X-ray absorptiometry, and bone metabolism markers. Similarly, the factors associated with the necessity of surgery and the progression of the narrowing of the joint space were evaluated.

**Results:**

Fifteen of the 44 hips required total hip arthroplasty (THA). A significant decrease was observed in the JSW from the first visit to the final follow-up. Changes in the JSW were associated with the length of band patterns, serum type 1 procollagen-N-propeptide (P1NP), and tartrate-resistant acid phosphatase 5b (TRACP-5b) during diagnosis. Additionally, bone metabolic markers tended to be associated with the length of the band pattern.

**Conclusions:**

SIF could cause joint space narrowing and hip OA. In addition to MRI findings as prognostic predictors of SIF, as previously described, bone metabolic markers were equally associated with changes in JSW, suggesting that these parameters could be useful in predicting the prognosis of SIF. Considering that bone metabolic markers trended to be associated with the length of band pattern, they might reflect the local severity.

## Background

Subchondral insufficiency fracture (SIF) of the femoral head has recently been recognized as a cause of the femoral head collapse, resulting in the degeneration of the hip joint, which is known to occur in association with osteonecrosis of the femoral head (ONFH) [1–3]. Although the precise prevalence of SIF is unknown, previous studies with histopathological re-evaluation showed that SIF was observed in 6.3% (460 of 7349) of patients preoperatively diagnosed with osteoarthritis (OA) and in 11.1% (41 of 369) of patients with ONFH [4]. Some cases of SIF have been reported to heal after conservative therapy, including rest, non-weight bearing, and traction [1, 5, 6], whereas other cases have been reported to undergo collapse necessitating surgery such as total hip arthroplasty (THA) and osteotomy [2, 3, 7, 8]. On the contrary, although there is evidence that the cause of primary OA is related to the changes in subchondral bone [9, 10], the influence of SIF on the degeneration of the hip joint and the prognostic factors related to joint degeneration among patients with SIF remain unclear.

SIF has been reported in adults of varying ages and activity levels [11–13]. Previous studies reported that the risk factors for THA were female sex [14], elderly onset [15], and length [16], location [17], and luminance of band pattern [18]. On the contrary, although bone fragility due to osteoporosis could be considered the most important cause of SIF, similar to vertebral body fractures [19], another study reported no differences in bone mass densitometry (BMD) between patients with and without (controls) SIF [20]. Bone strength is determined by bone mass, geometry, and quality, including bone turnover, microarchitecture, and the degree and distribution of mineralization [21]. Among these, one review article reported a variation in the levels of bone turnover markers throughout the course of fracture repair, which was dependent on the size of the fracture and the healing time [22]. Therefore, evaluating whether and how bone turnover markers would affect the pathology of SIF would be of significant interest.

Hence, the objectives of this study were (1) to investigate the natural history of joint space width after the occurrence of SIF and (2) to investigate the associations between joint space narrowing and bone metabolic markers as well as magnetic resonance imaging (MRI). The hypotheses of this study were as follows: (1) SIF could induce joint space narrowing and hip OA, and (2) bone metabolic abnormalities and MRI findings could predict the prognosis and reflect the severity of SIF.

## Patients and methods

**Fig. 1:**
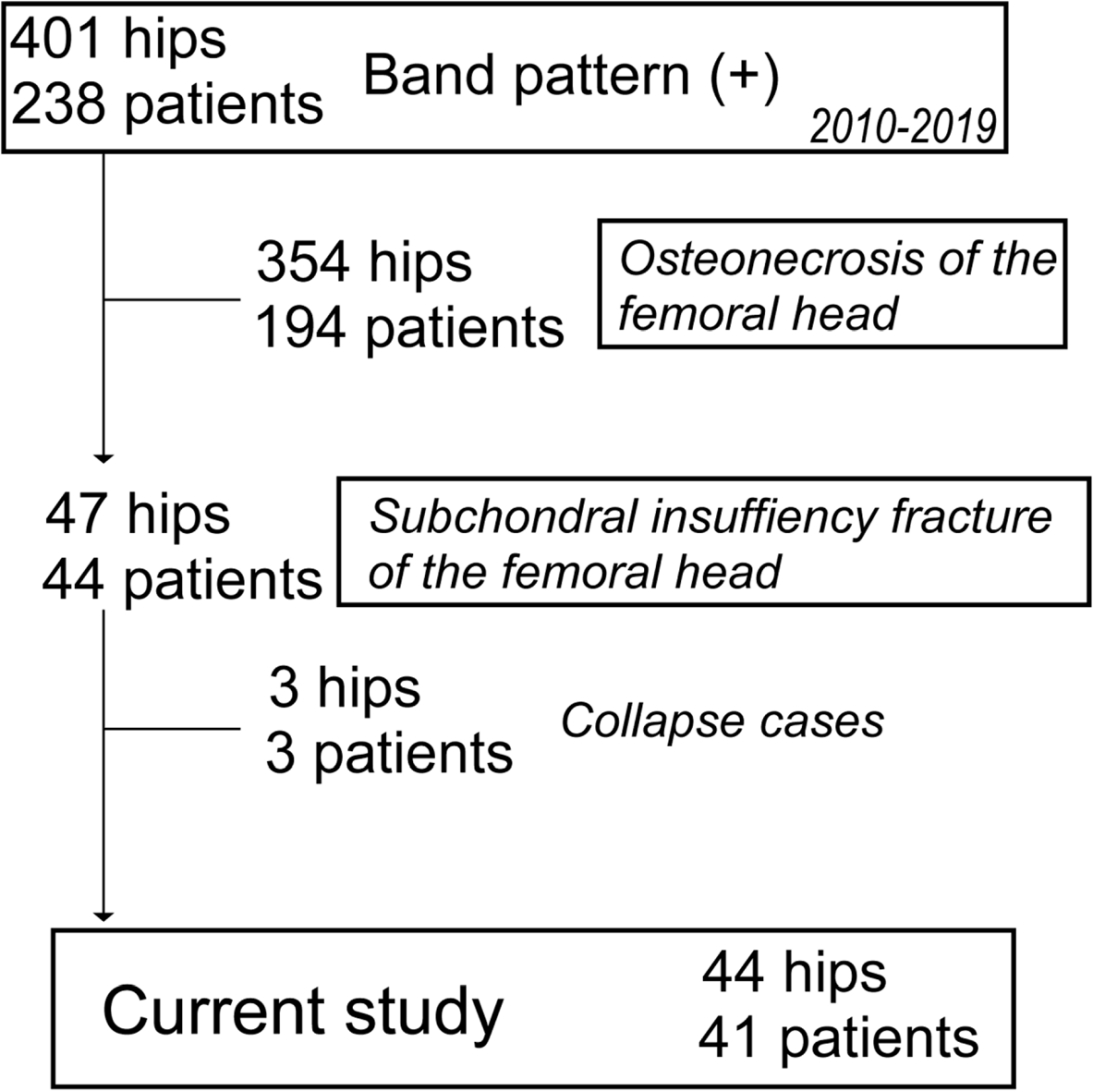
Flow diagram of study subjects

The Institutional Review Board approved this retrospective study (# 015-0206). In total, 238 patients (401 hips) who showed a band pattern of the femoral heads on MRI visited Hokkaido University Hospital from January 2010 to December 2019. Among these, SIF was diagnosed based on several published criteria [2, 23, 24]: hip pain that manifested without any apparent history of trauma; radiographs that were normal or indicated a collapse of the femoral head, joint space narrowing, and/or a linear patchy sclerotic area in the superior portion of the femoral head; a bone marrow edema pattern in the femoral head and/or neck on MRI; and a subchondral low signal-intensity band on T1-weighted MRI that was convex to the articular surface and parallel to the subchondral bone end-plate. We distinguished between SIF and ONFH via gadolinium-enhanced MRI. Forty-seven hips in 44 patients (male 10, female 34) were diagnosed with SIF. In this study, 3 hips in 3 patients who demonstrated rapid collapse and joint destruction, including that of the acetabular (AC), were excluded (Fig. 1). Power analysis was performed to detect a 10% difference in the survival rate between patients with and without MRI positive findings (intensity change area) with a power of 80% at a significance level of 0.05. Thirty-three subjects were needed, and we could recruit a sufficient number of patients for this study.

Among those diagnosed with SIF, patients who could be diagnosed within 3 months after hip pain were supervised to avoid weight-bearing with crutches for 6 weeks [25] and were treated on an outpatient basis every 2 weeks. Patients with late diagnosis or poor compliance could not be initially treated with conservative therapy.

Data on patient demographics, including age, sex, and body mass index (BMI); the period from onset to the first visit; history of corticosteroid intake or alcohol abuse; and medical history of osteoporosis drug intake were collected from their medical records. Data regarding the possible treatment of patients with conservative therapy and the requirement of THA within the follow-up period were similarly collected. The indication of THA was persistent pain and disability of daily life activities regardless of nonsteroidal anti-inflammatory medications. Alcohol abuse was defined as the consumption of more than 400 mL of alcohol per week, which is known to be a significant risk factor for osteonecrosis of the femoral head [26].

**Fig. 2:**
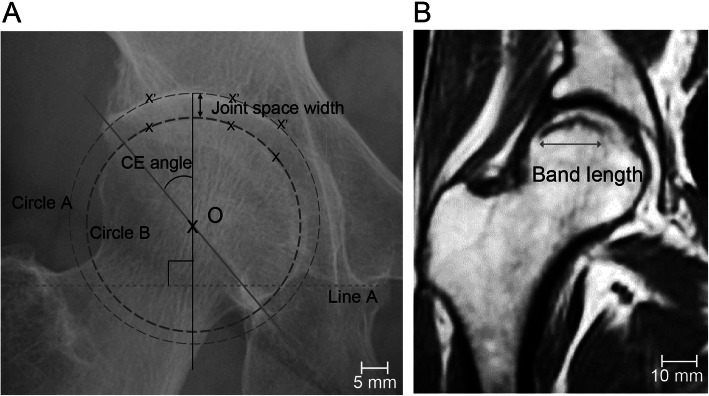
Radiological evaluation. **a** The radiographic indices used for the evaluation of the hip are shown. O = center of the femoral head; Line A = line between the teardrops on both sides; Circle A = circle passing through three points set arbitrarily in the acetabular joint surface; Circle B = circle passing through three points set arbitrarily in the femoral head. Scale bar, 5 mm. **b** T1-weighted magnetic resonance imaging (MRI) image illustrating the method used to measure the length of the low-intensity band. Scale bar, 10 mm. CE, center-edge

Radiographs were taken using a similar technique throughout the study period; a standardized position of the beam and radiographic penetration were adopted. The radiographs of all patients were assessed using a picture archiving and communication system (PACS) on the anteroposterior (AP) radiographs. In this study, the center-edge (CE) angle at the first visit and longitudinal joint space width (JSW) were investigated (Fig. 2a). In JSW analysis, concentric circles passing through three points set arbitrarily in the AC joint surface and the femoral head were drawn (circle A and circle B in Fig. 2a). The distance between the intersection of each circle and the line that runs through the center of the femoral head (O in Fig. 2a) perpendicular to the line between the bilateral teardrops (line A in Fig. 2a) was measured. The interobserver variability in the JSW between two observers (YK and TD) was 0.768.

In this study, the MRI examinations were performed using a 1.5-T system under 5-mm slice thickness within 1 week after the first visit to hospital. The T1- and T2-weighted spin-echo images and short tau inversion recovery (STIR) images on the coronal and axial (and/or oblique axial: paralleling the femoral neck axis) planes were available in all cases. The band lengths were measured at the slice wherein the longest band was detected on the T1-weighted MRI (on the coronal plane), as previously described [16] (Fig. 2b). The interobserver variability in the band length between two observers (YK and TD) was 0.836.

Fasting blood samples were obtained to examine the biochemical markers of osteoporosis-related bone turnover, including the levels of intact type 1 procollagen-N-propeptide (P1NP) and tartrate-resistant acid phosphatase 5b (TRACP 5b). Because type I collagen-derived peptides, such as CTX-1 (cross-linked C terminal telopeptides of type I collagen) and NTX-1 (cross-linked N-telopeptide of type I collagen) are excreted through the kidneys, they can be affected by renal dysfunction; therefore, this study evaluated the serum levels of P1NP and TRACP-5b. Serum P1NP level was measured using electrochemiluminescence immunoassay, whereas serum TRACP-5b level was measured using enzyme immunoassay (SRL, Inc. Tokyo). Areal BMD in the lumbar spine (LS, L2–L4) and femoral neck was assessed by dual-energy X-ray absorptiometry (DXA; Discovery A, Hologic Japan, Inc., Tokyo, Japan). Bone turnover markers and BMD were investigated during the definite diagnosis following the acquisition of MRI images.

Chi-squared or independent *t* tests were used to compare the differences between patients who required THA and those treated conservatively and between patients who could comply with the weight-bearing limitation and those who could not. Cox regression analysis was performed to identify the risk factors for THA. Linear regression models were adjusted for age, sex, BMI, and anti-osteoporosis therapy; in addition, the period from onset to diagnosis was established to determine the associations between changes in the JSW, band length, and bone metabolic markers. All statistical analyses were performed using SPSS Statistics version 23.0 (IBM Corporation, Armonk, NY); values of *p* less than 0.05 were considered statistically significant.

## Results

The demographics and clinical data of the patients are summarized in Table 1. In total, 18 of 44 hips could complete the weight-bearing limitation for 6 weeks. Six hips that could not complete the weight-bearing limitation were the late diagnosis cases. Of 7 patients who had undergone anti-osteoporosis therapy, one patient was treated monthly with minodronic acid and the others were treated by active vitamin D3. Fifteen (14 patients) of 44 hips required THA. The mean period from the diagnosis of SIF to THA was 10.5 months (range; 2–54 months). Ten hips (9 patients) developed hip dysplasia (CE < 20 degrees).

**Table 1:** Patient demographics

41 patients, 44 hips	
Age, years	61.6 (2.3)
Sex, male to female	8:33
Body mass index, kg/m^2^	25.5 (0.8)
Period from onset to first visit, months	2.2 (0.4)
Follow-up period, months	26.6 (2.3)
Glucocorticoid use, cases	9
Alcohol abuse, cases	6
Anti-osteoporosis therapy, cases	7
Weight-bearing limitation, hips	18
Total hip arthroplasty, hips	15

Data are represented as the mean (standard error of the mean)

**Fig. 3:**
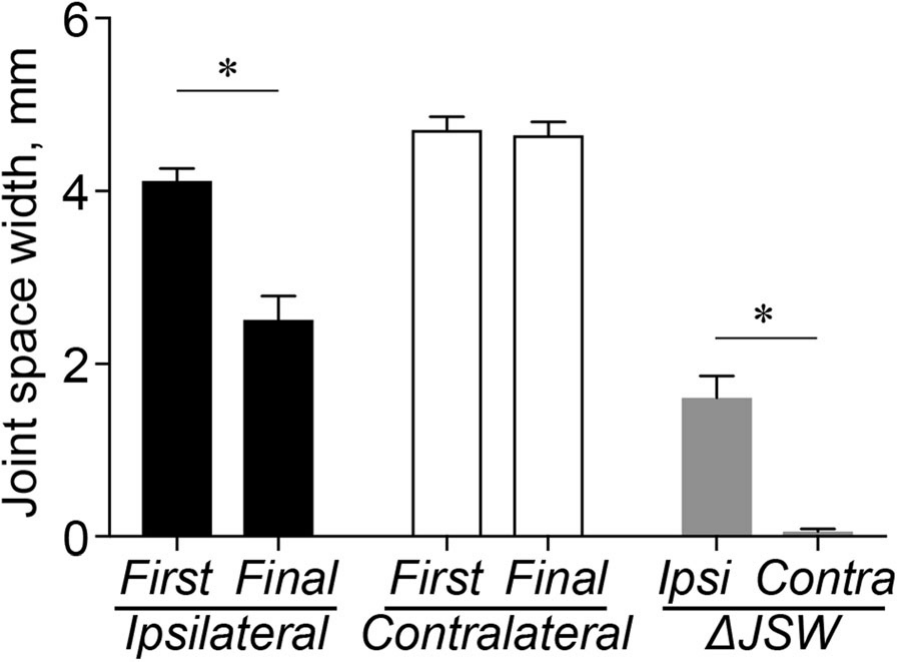
Comparisons of joint space width. Mean joint space width and change in joint space width from the first visit to the final follow-up in the ipsilateral and contralateral sides. Ipsi, ipsilateral; Contra, contralateral; JSW, joint space width. Asterisks indicate *P* < 0.05

**Fig. 4:**
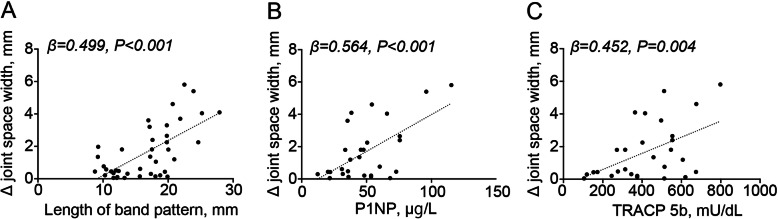
Association with the changes in joint space width. Scatter plot of changes in joint space width versus **a** length of band pattern, **b** type 1 procollagen-N-propeptide, and **c** tartrate-resistant acid phosphatase 5b. P1NP, type 1 procollagen-N-propeptide; TRACP 5b, tartrate-resistant acid phosphatase 5b

**Fig. 5:**
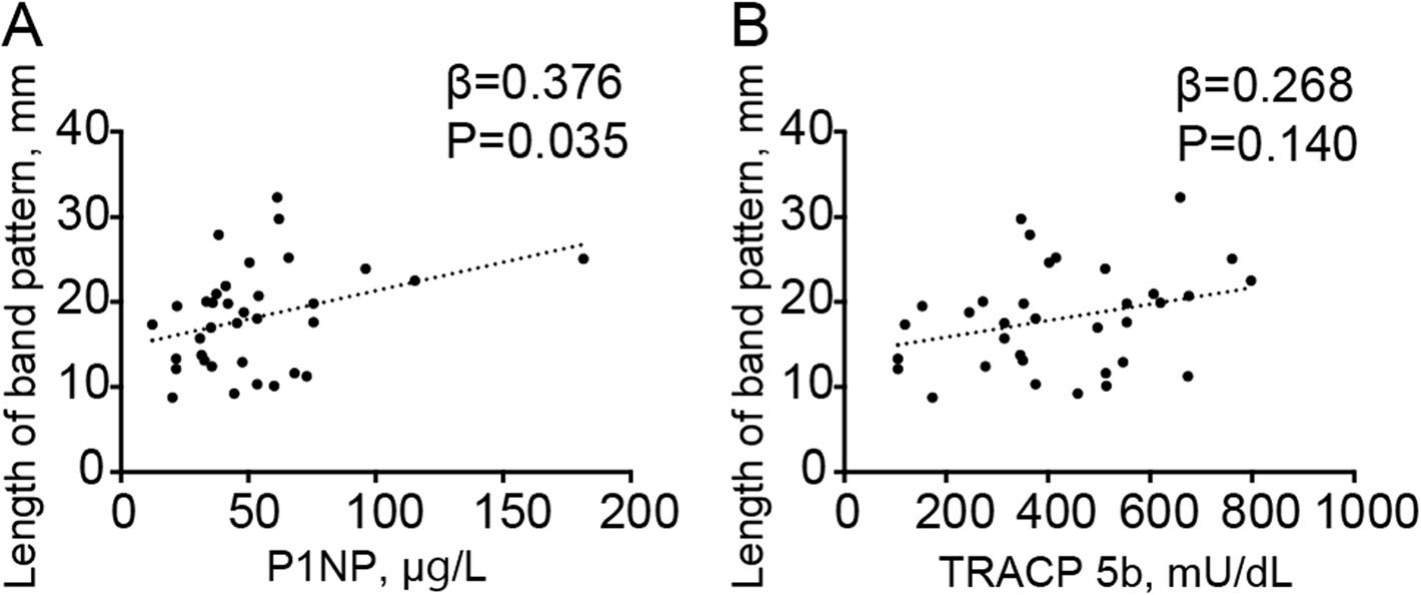
Association between the length of band pattern and bone metabolic markers. Scatter plot of the length of band pattern versus **a** type 1 procollagen-N-propeptide and **b** tartrate-resistant acid phosphatase 5b. P1NP, type 1 procollagen-N-propeptide; TRACP 5b, tartrate-resistant acid phosphatase 5b

The JSW in the ipsilateral side demonstrated a significant decrease from the first visit to the final follow-up (*P* < 0.001) (Fig. 3). No significant difference was observed in the JSW from the first visit to the final follow-up on the contralateral side. The changes in the JSW on the ipsilateral side were associated with the length of the band pattern (*β* = 0.499, *P* < 0.001) (Fig. 4a), serum P1NP (Fig. 4b) (*β* = 0.564, *P* < 0.001), and TRACP 5b (Fig. 4c) (*β* = 0.452, *P* = 0.004). Additionally, the serum P1NP and TRACP 5b levels tended to be associated with the length of the band pattern (*β* = 0.376, *P* = 0.035, and *β* = 0.268, *P* = 0.140, respectively) (Fig. 5a, b).

The comparisons of clinical and radiological findings between patients who could complete the weight-bearing limitation therapy (WB limitation) and could not (non-WB limitation) are summarized in Table 2. WB limitation group showed a younger age, shorter period from pain onset to visit, lower ratio of THA, and smaller changes in JSW than non-WB limitation group.

**Table 2:** Comparisons between patients who could complete weight-bearing limitation and those who could not

	WB limitation (18 hips)	Non-WB limitation (26 hips)	***P*** value
Male to female	5:13	3:23	0.170
Age, years	53.8 (4.3)	67.2 (2.0)	***0.003***
BMI, kg/m^2^	26.4 (1.8)	25.1 (1.1)	0.525
Period from pain onset to visit, months	1.1 (0.2)	2.5 (0.4)	***0.008***
Osteoporosis therapy, case (%)	1 (5.6%)	6 (22.2%)	0.118
Total hip arthroplasty, case (%)	3 (16.7%)	12 (46.2%)	***0.042***
Radiography findings			
JSW at the first visit, mm	4.15 (0.19)	4.25 (0.21)	0.785
Changes in JSW, mm	1.12 (0.36)	2.24 (0.36)	***0.039***
Center-edge angle, degree	25.9 (1.6)	24.2 (1.4)	0.428

Data are represented as the mean (standard error of the mean)

*WB* weight-bearing, *BMI* body mass index, *JSW* joint space width, *AC* acetabulum

**Fig. 6:**
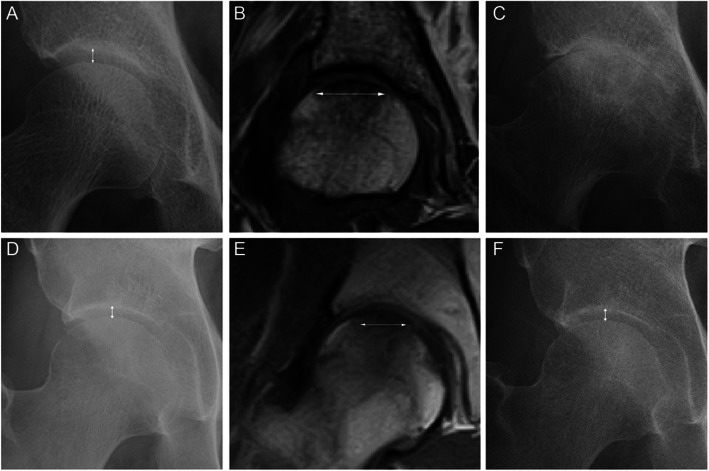
Longitudinal radiological images in patients who underwent total hip arthroplasty and those who did not. **a** Anteroposterior (AP) radiograph of the hip at the first visit in a 68-year-old female with hip pain. White arrow represents joint space width. **b** Coronal T1-weighted magnetic resonance image (MRI) of the hip at the same week. White arrow represents length of low-intensity band pattern in the femoral head. **c** AP radiograph of the hip at the final follow up. Joint space narrowing progressed, and she underwent total hip arthroplasty. **d** AP radiograph of the hip at the first visit in a 47-year-old male with hip pain. White arrow represents joint space width. **e** Coronal T1-weighted MRI of the hip at the same week. White arrow represents length of low-intensity band pattern in the femoral head. **f** AP radiograph of the hip at the final follow-up (3 years after the first visit). Patient demonstrated no progression in joint space narrowing

The comparisons of the clinical findings, image evaluation, and bone metabolic markers between patients who underwent THA (THA group) and who did not undergo THA (non-THA group) are summarized in Table 3. The non-THA group was younger (*P* = 0.028) and had a shorter period from pain onset to visit (*P* = 0.002) and higher ratio of weight-bearing limitation (*P* = 0.042) than the THA group. No significant differences in JSW and CE angle at the first visit were observed between the two groups. The THA group exhibited a more significant change in JSW from the first visit to the final follow-up than the non-THA group (*P* < 0.001) (Fig. 6a, c, d, f). In addition, the THA group exhibited longer band length (*P* < 0.001) (Fig.6b, e), a higher ratio of the existence of band over the edge of AC (*P* = 0.006), and a more significant intensity change in AC (*P* < 0.001) than the non-THA group. Although no significant differences were observed in BMD between both groups, the THA group exhibited higher P1NP (*P* = 0.002) and TRACP 5b (*P* = 0.001) levels than the non-THA group.

**Table 3:** Comparisons between patients who received total hip arthroplasty and did not

	THA (15 hips)	Non-THA (29 hips)	***P*** value
Male to female	3:12	5:24	0.822
Age, years	69.6 (2.2)	57.8 (3.1)	***0.014***
BMI, kg/m^2^	24.9 (1.5)	26.0 (1.4)	0.593
Osteoporosis therapy, case (%)	2 (13.3%)	5 (17.2%)	0.737
Period from pain onset to diagnosis, months	3.8 (1.0)	1.3 (0.2)	***0.002***
Weight-bearing limitation, case (%)	3 (20.0%)	15 (51.7%)	***0.042***
Radiography findings			
JSW at the first visit, mm	4.26 (0.28)	4.18 (0.18)	0.793
Changes in JSW, mm	3.79 (0.36)	0.75 (0.15)	***< 0.001***
Center-edge angle, degree	25.3 (1.6)	24.7 (1.4)	0.786
MRI findings			
Band length, mm	23.3 (1.1)	14.2 (0.7)	***< 0.001***
Band over the edge of AC, case (%)	10 (66.7%)	7 (24.1%)	***0.006***
Intensity change of AC, case (%)	14 (93.3%)	6 (20.7%)	***< 0.001***
YAM, %			
Lumbar	93.9 (4.8)	92.6 (3.6)	0.824
Femoral neck	80.2 (2.7)	82.0 (2.3)	0.632
Bone metabolic marker			
P1NP, μg/ml	73.0 (11.1)	39.7 (3.7)	***0.002***
TRACP 5b, mU/dL	549.7 (40.6)	342.4 (38.1)	***0.001***

Data are represented as the mean (standard error of the mean)

*THA* total hip arthroplasty, *BMI* body mass index, *JSW* joint space width, *AC* acetabulum, *YAM* young adult mean, *P1MP* intact type 1 procollagen-N-propeptide, *TRACP 5b* tartrate-resistant acid phosphatase 5b

**Fig. 7:**
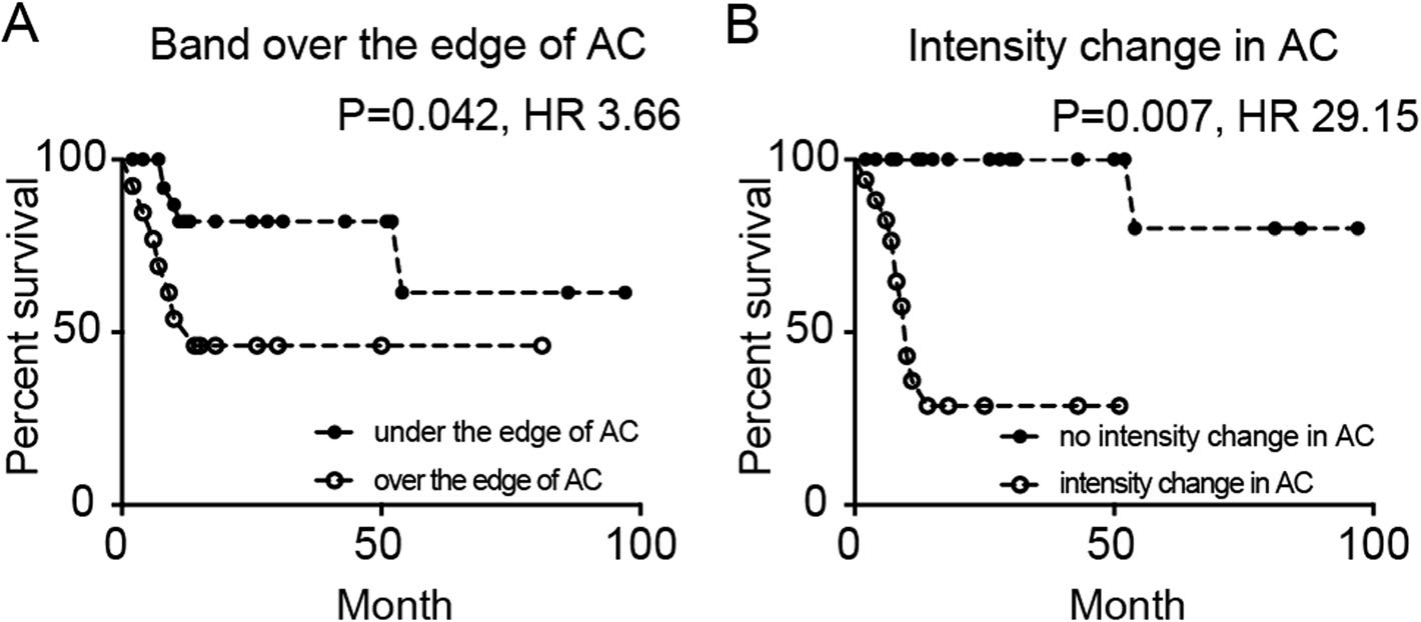
Kaplan–Meier curves for the requirement of total hip arthroplasty. Kaplan–Meier curves for the requirement of total hip arthroplasty (THA) between **a** patients with and without intensity changes in the acetabulum and **b** patients in whom the band pattern ended and was under the edge of the acetabulum. AC, acetabulum; HR, hazard ratio

In the univariate analyses, age, period from pain onset to visit, MRI findings, and bone metabolic markers were identified as predictors of THA. Furthermore, MRI findings were identified as the potential predictors of THA in a Cox proportional hazard model adjusted for age, sex, BMI, anti-osteoporosis therapy, and the period from pain onset to visit (Table 4) (Fig. 7).

**Table 4:** Univariate and multivariate Cox-regression analysis for the predictors of total hip arthroplasty in patients with subchondral insufficient fracture of the femoral head

Variables	Univariate analysis			Multivariate analysis		
	*P* value	HR	95% CI	*P* value	HR	95% CI
Sex	0.493	1.56	0.438–5.557			
Age	0.020	1.07	1.011–1.129			
BMI	0.659	0.98	0.889–1.077			
Osteoporosis therapy	0.665	0.72	0.161–3.208			
Period from onset to diagnosis	0.021	1.28	1.038–1.578			
Weight-bearing limitation	0.107	0.35	0.099–1.251	0.727	0.77	0.179–3.324
MRI findings						
Band length	< 0.001	1.30	1.152–1.455	0.001	1.29	1.116–1.495
Band over the edge of AC	0.014	3.87	1.317–11.375	0.042	3.66	1.049–12.803
Intensity change of AC	0.001	28.85	3.677–226.286	0.007	29.15	2.517–337.594
Bone metabolic marker						
P1NP, μg/ml	0.018	1.013	1.002–1.024	0.148	1.01	0.997–1.023
TRACP 5b, mU/dL	0.007	1.005	1.001–1.008	0.114	1.00	0.999–1.008

Multivariate analyses were adjusted for sex, age, BMI, anti-osteoporosis therapy, and period from pain onset

*HR* hazard ratio, *CI* confidence interval, *BMI* body mass index, *AC* acetabulum, *P1NP* intact type 1 procollagen-N-propeptide, *TRACP 5b*: tartrate-resistant acid phosphatase 5b

## Discussion

This study exhibited that JSW significantly decreased from the first visit to the final follow-up and approximately one third of patients progressed to THA; this suggest that SIF could cause hip OA. Since some cases of SIF resolved by weight-bearing limitation [2, 27, 28], nonoperative treatment would be the first choice in all patients. Although the multivariate Cox-regression analysis in this study showed that weight-bearing limitation could not prevent THA, it could reduce the progression of joint space narrowing. Additionally, considering that age and the period from pain onset to visit were significantly different between the WB and non-WB limitation groups, the association with THA, early detection, and treatment are important for SIF. There might be some cases, such as older patients with impaired vision or low balance activity, who require treatment on an inpatient basis, rather than an outpatient basis.

The study findings showed that bone metabolic markers and the length of band patterns were associated with the changes in JSW, suggesting that these parameters could be predictors of poor progression in patients with SIF. The MRI findings of this study were consistent with those of previous reports regarding the association of clinical outcome and MRI [16, 25], suggesting that MRI findings, including the length of band pattern and location of intensity change during diagnosis, could be prognostic predictors as well as highly sensitive indicators for SIF investigation. In addition, serum P1NP and TRACP-5b were associated with joint space narrowing, suggesting that the levels of bone metabolic markers might be predictors for OA among patients with SIF. Considering that serum P1NP and TRACP-5b tended to be associated with the length of band pattern, bone metabolic markers might reflect the severity of fracture. On the contrary, because this study investigated only serum P1NP and TRACP-5b, future study should address the mechanism underlying the association of other bone turnover markers, such as bone specific alkaline phosphatase, osteocalcin, CTX-1, and NTX-1 with joint space narrowing in patients with SIF.

While numerous groups reported the occurrence of SIF in patients with osteoporosis [1, 2, 6, 7], this study showed that mean BMD did not fulfill the diagnostic criteria for osteoporosis. On the contrary, in some reports on young adults and adolescents, the activity levels did not correspond to a high occurrence of SIF [12, 29]. Therefore, the mechanisms of occurrence and joint space narrowing associated with osteoporosis in patients with SIF remain unclear. Contrary to findings in previous reports, the mean CE angle observed in this study did not fulfill the criteria for a dysplastic hip (CE < 20°) [20]. In addition, the CE angle in this study was not associated with the changes in JSW and clinical outcomes. A recent study reported that SIF with pre-collapse was associated with bony deformities and lateral labral tears [30]. Therefore, the instability of the hip joint induced by abnormal morphologies, such as dysplasia and femoroacetabular impingement, might affect the occurrence and prognosis of SIF. Although this study did not obtain radial MRI or investigate AC labral tears, future studies should address the association between labral tears and the prognosis of SIF.

There are some limitations to this study. First is the timing of the occurrence of SIF. Since SIF usually occurs without a history of trauma, it is challenging to clarify the accurate timing of the occurrence. The second is the method of JSW measurement. This study investigated the JSW following the technique shown in Fig.1a, and we believed that this method was highly reproducible. However, future studies should address the JSW measurement via computer-based radiographic quantification. The third is the short follow-up duration (mean of 25.4 months) of JSW. Therefore, future studies are warranted to verify the relation of prognostic predictors to the changes in JSW in longer follow-up periods.

## Conclusion

In conclusion, SIF could cause joint space narrowing and hip OA. In addition to MRI findings as prognostic predictors of SIF, as previously described, bone metabolic markers were equally associated with the changes in JSW, suggesting that these parameters could be useful in predicting the prognosis of SIF. Considering that bone metabolic markers tended to be associated with the length of band pattern, they might reflect the local severity.

## Data Availability

The datasets used and/or analyzed during the present study are available from the corresponding author on reasonable request.

## Abbreviations

AC: Acetabular
AP: Anteroposterior
BMD: Bone mineral density
BMI: Body mass index
CE: Center-edge
CTX: Cross-linked C terminal telopeptides of type I collagen
JSW: Joint space width
MRI: Magnetic resonance imaging
NTX: Cross-linked N-telopeptide of type I collagen
OA: Osteoarthritis
ONFH: Osteonecrosis of the femoral head
PACS: Picture archiving and communication system
P1NP: Type 1 procollagen-N-propeptide
SIF: Subchondral insufficiency fracture of the femoral head
THA: Total hip arthroplasty
RACP-5b: Tartrate-resistant acid phosphatase 5b
